# Scutellarin inhibits proliferation and invasion of hepatocellular carcinoma cells via down‐regulation of JAK2/STAT3 pathway

**DOI:** 10.1111/jcmm.14169

**Published:** 2019-01-29

**Authors:** Kang Liu, Tian Tian, Yi Zheng, Linghui Zhou, Cong Dai, Meng Wang, Shuai Lin, Yujiao Deng, Qian Hao, Zhen Zhai, Zhijun Dai

**Affiliations:** ^1^ Department of Breast Surgery, Guangzhou Women and Children’s Medical Center Guangzhou Medical University Guangzhou China; ^2^ Department of Hepatobiliary Surgery The First Affiliated Hospital of Xi'an Jiaotong University Xi'an Shaanxi China; ^3^ Department of Oncology The Second Affiliated Hospital of Xi’an Jiaotong University Xi'an Shaanxi China

**Keywords:** cytoskeleton, EMT, hepatocellular carcinoma, JAK2/STAT3, scutellarin

## Abstract

The prognosis of hepatocellular carcinoma (HCC) is poor because of high incidence of recurrence and metastasis. JAK/STAT signalling pathway regulates cell proliferation, apoptosis, differentiation and migration and epithelial‐mesenchymal transition (EMT) is also considered to contribute to invasion and metastasis of epithelial malignant tumours. Scutellarin is an active component found in many traditional Chinese herbs and has been regularly used in anti‐inflammatory and antitumour medicine. This study aimed to identify the effect of scutellarin and its possible mechanism of action in HCC cells. Proliferation, colony‐forming, apoptosis and cell migration assays were used to examine the effect of scutellarin on HCC cells. Quantitative real‐time PCR and Western blotting were performed to study the molecular mechanisms of action of scutellarin. Light and electron microscopy and immunofluorescence analysis were performed to study the effect of scutellarin on cellular mechanics. We show that scutellarin potentially suppresses invasiveness of HepG2 and MHCC97‐H cells in vitro by remodelling their cytoskeleton. The molecular mechanism behind it might be the inhibition of the EMT process, which could be attributed to the down‐regulation of the JAK2/STAT3 pathway. These findings may provide new clinical ideas for the treatment of liver cancer.

## 1 INTRODUCTION

Hepatocellular carcinoma (HCC) is one of the five most commonly occurring cancers^1^ and the second leading cause of all cancer‐related deaths worldwide.^2^ The treatment of liver cancer has long been in the process of development. However, the prognosis of liver cancer treatment is still poor because of its recurrence and metastasis. Invasion and migration are regarded as the two most significant biological characteristics of malignant tumours.

The Janus kinase/signal transducer and activator of transcription (JAK/STAT) pathway is considered to be one of the most significant and active signalling pathways in cells that transduce signals downstream to cytokines, growth factors and hormones.^3^ It has been verified that inhibition of JAK2/STAT3 pathway could suppress migratory and invasive potential in some cancers. Epithelial‐mesenchymal transition (EMT) refers to the biological process by which the action of signalling molecules mediates the transformation of epithelial cells into mesenchymal cells.^4^

Scutellarin is an effective component found in many traditional Chinese herbs. In recent years, many studies have shown that scutellarin shows antitumour effects in colon, breast and tongue cancers and acts by different mechanisms including promoting apoptosis and inhibiting invasion.^5^, ^6^, ^7^, ^8^, ^9^ We investigated whether scutellarin could inhibit HCC cells and the possible mechanisms of its action.

## 2 MATERIALS AND METHODS

Human HCC cell lines were purchased from Shanghai Cell Biological Institute of the Chinese Academy of Science and cultured in DMEM supplemented with 10% foetal calf serum and 1% penicillin–streptomycin in a humidified atmosphere of 5% CO_2_ and 95% air at 37°C. Scutellarin was purchased from Melone Pharmaceutical Co., Ltd. Scutellarin was dissolved in DMSO for all experiments. All other reagents, if not specifically noted, were purchased from Sigma.

MTT assay was conducted to evaluate the effect of scutellarin on proliferation of HCC cells. Colony‐forming assay was conducted to detect the colony‐forming ability of HCC cells after treated with scutellarin. Apoptosis assay was conducted to evaluate the effect of scutellarin on apoptosis of HCC cells. Cell migration assay was performed to evaluate the effect of scutellarin on migration of HCC cells. Quantitative real‐time PCR and Western blot analysis were performed to detect mRNA expression and protein expression of JAK2, STAT3 and EMT‐related genes in HCC cells. Light and electron microscopy and immunofluorescence were applied to visualize the cytoskeleton in HCC cells.

The data are presented as mean ± SD. Intra‐group comparisons were made by employing Student's *t* test or an analysis of variance. All statistical analyses were performed with SPSS version 17.0 and all figures were generated using GraphPad Prism 5.01. The significance level was set at *P* < 0.05.

## 3 RESULTS

**Figure 1:**
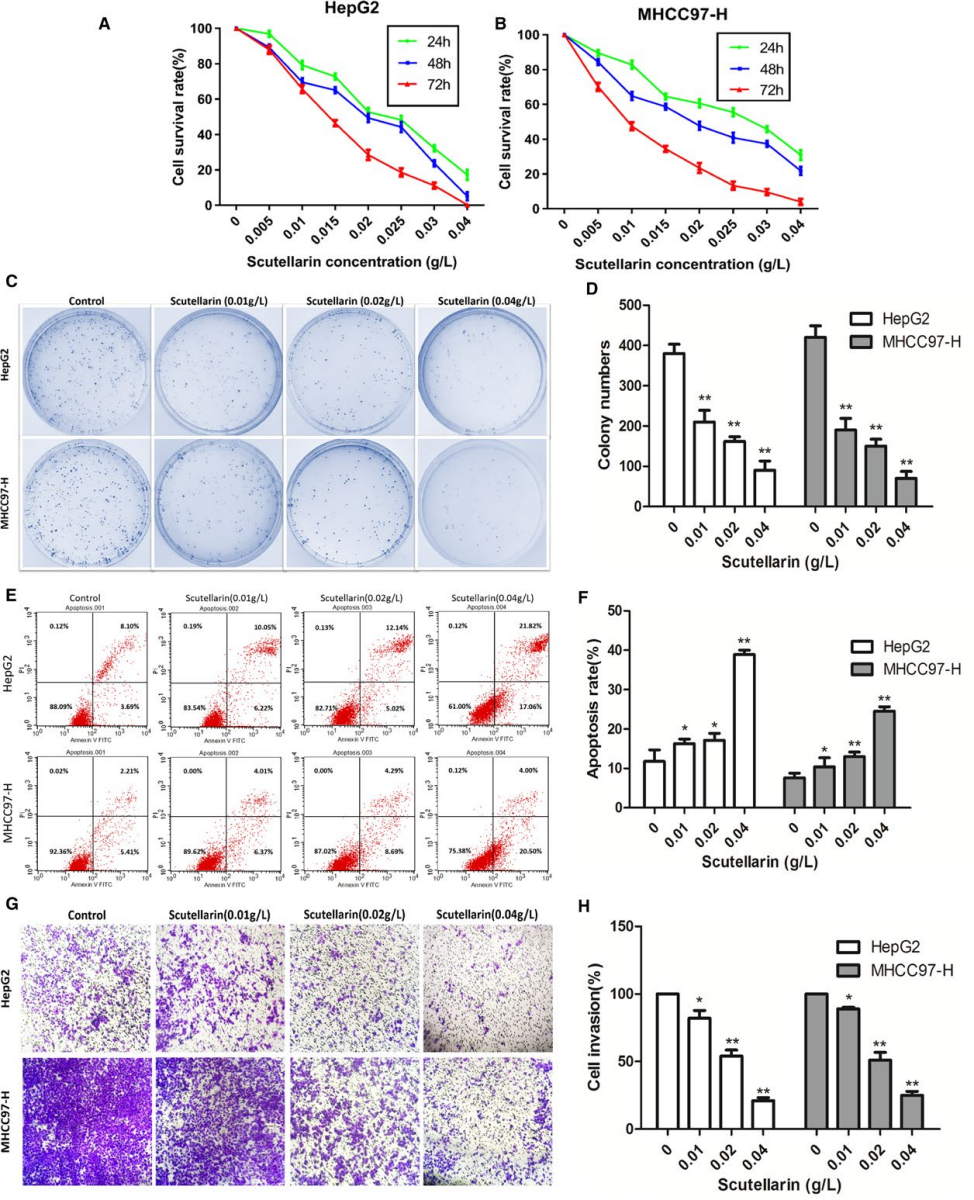
The effect of scutellarin exerted on hepatocellular carcinoma cells. A, HepG2 cell viability was measured by MTT assay. B, MHCC97‐H Cell viability was measured by MTT assay. Values were represented as mean ± SD of three independent experiments performed in triplicate. C, Cell colony‐forming capacity was measured by Plate colony‐forming assay. Colony formation was photographed and counted under a 100× magnification. D, Quantification of colony‐forming assay. E, Cell apoptosis was detected by flow cytometry. F, Quantification of apoptosis assay. G, Cell invasion ability was assessed by transwell invasion assay. H, Quantification of invasion assay. *P < 0.05 and **P < 0.01 compared with the control group. Data are presented as the mean ± SD of three separate experiments. SD: standard deviation

As shown in Figure 1A and 1. The proliferation of HepG2 and MHCC97‐H cells was inhibited by scutellarin in a dose‐ and time‐dependent manner. HepG2 and MHCC97‐H cells treated with different concentrations of scutellarin (0.01, 0.02 and 0.04 g/L) showed a significant decrease in colony numbers compared to the untreated control cells (Figure 1C and 1). As shown in Figure 1E and 1, compared with the untreated control groups, higher apoptosis rates were observed in HepG2 and MHCC97‐H cells exposed to different concentrations of scutellarin (0.01, 0.02 and 0.04 g/L). Next, we assessed the invasive capability of HCC cells by transwell invasion assay. Figure 1G shows that scutellarin treatment reduced the invasiveness of both HepG2 (low invasiveness) and MHCC97‐H (high invasiveness) cells in a concentration‐dependent manner. Similar trend was also noted in MHCC97‐H cells, wherein the invasive potential was suppressed by 19.38%, 49.87% and 79.69% respectively upon scutellarin treatment (Figure 1H).

**Figure 2:**
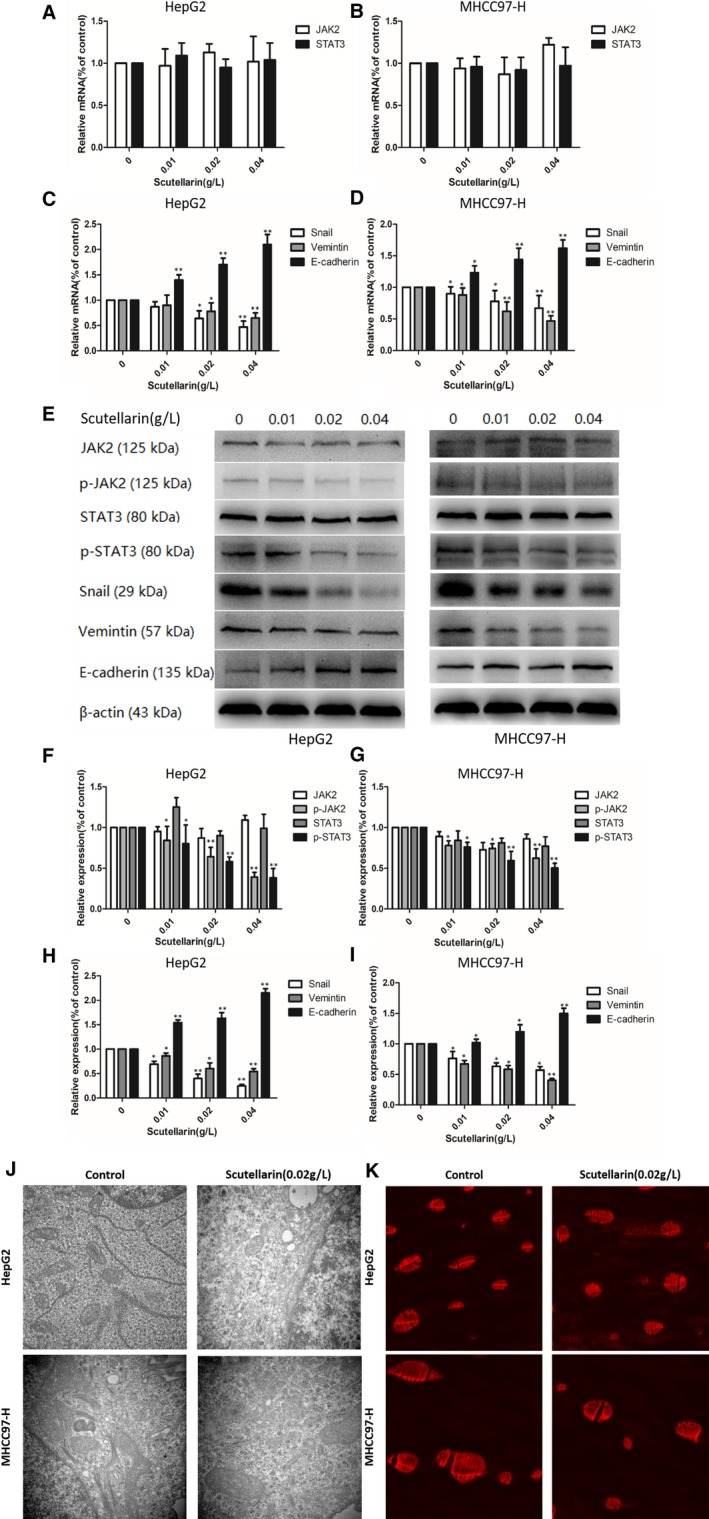
Molecular mechanism and biomechanics mechanism of scutellarin influenced hepatocellular carcinoma cells. A, The effects of scutellarin on the expression levels of JAK2 and STAT3 mRNA in HepG2 cell lines. B, The effects of scutellarin on the expression levels of JAK2 and STAT3 mRNA in MHCC97‐H cell lines. C, The effects of scutellarin on the expression levels of EMT‐related molecules in HepG2 cell lines. D, The effects of scutellarin on the expression levels of EMT‐related molecules in MHCC97‐H cell lines. E, Hepatocellular carcinoma cells were treated with scutellarin (0, 0.01, 0.02 and 0.04 g/L) for 24 h and then subjected to Western blotting to analyse the protein levels of JAK2, STAT3 and EMT‐related molecules. F, Quantification of the protein levels of JAK2 and STAT3 mRNA in HepG2 cell lines. G, Quantification of the protein levels of JAK2 and STAT3 mRNA in MHCC97‐H cell lines. H, Quantification of the protein levels of EMT‐related molecules in HepG2 cell lines. I, Quantification of the protein levels of EMT‐related molecules in MHCC97‐H cell lines. J, Cytoskeleton in hepatocellular carcinoma cells was observed after treating with scutellarin under electron microscopy (×40 000). K, The visualization of F‐actin microfilaments in hepatocellular carcinoma cells under fluorescence microscope (×400). *P < 0.05 and **P < 0.01 compared with the control group. Data are presented as the mean ± SD of three separate experiments

As shown in Figure 2A and 2, the mRNA levels of *JAK2* and *STAT3* did not change significantly with increase in scutellarin concentration. However, we observed an increase in the mRNA expression of *E‐cadherin*, which is representative of epithelial cell lineage, while the transcript levels of *Snail* and *Vimentin* genes expressed in the mesenchymal cells decreased **(**Figure 2C and 2). Western blotting was performed to investigate the effect of scutellarin treatment on the protein expression of JAK2, STAT3 and EMT‐related proteins in HCC cells (Figure 2E). Quantitation of protein amounts indicated that while the levels of JAK2 and STAT3 showed no significant changes upon treatment with different concentrations of scutellarin, the levels of phosphorylated JAK2 and STAT3 decreased significantly (Figure 2F and 2). As shown in Figure 2H and 2, with an increase in scutellarin concentration, the levels of E‐cadherin increased and the protein levels of snail and vimentin decreased, which was consistent with the changes in mRNA expression.

Cytoskeletal elements play important roles in cell mobility and invasiveness.^10^ To determine if scutellarin could alter cytoskeleton in HCC cells, we used electron microscopy to observe the cytoskeleton in HepG2 and MHCC97‐H cells after treating these cells with 0.02 g/L scutellarin. As shown in Figure 2J, the cytoskeletal structures were altered in HepG2 and MHCC97‐H cells after treatment with scutellarin. As scutellarin alters the cytoskeleton in HepG2 and MHCC97‐H cells, we next examined the F‐actin in these cells, because it is an important constituent of cytoskeleton. We used phalloidin staining to visualize the F‐actin microfilaments in HCC cells. As shown in Figure 2K, immunofluorescence analysis indicated that the shape of HepG2 and MHCC97‐H cells showed marked changes after treatment with 0.02 g/L scutellarin.

## 4 DISCUSSION

JAK2 is a member of the JAK family of protein tyrosine kinases, which performs diverse functional roles in carcinogenesis.^11^ STAT3 is considered to be a critical transcription activator for cell survival‐related genes and cell cycle and its phosphorylation could be linked to HCC tumour progression.^12^ Many studies have reported that the activation of JAK2/STAT3 pathway contributes to progression of EMT in various cancer cells.^13^, ^14^

Cancer cells break down cell‐cell contacts and remodel cell‐matrix adhesion sites to detach from the primary site and to invade into the surrounding tissue. These processes are also observed in various non‐pathological conditions, such as EMT which is observed in developmental stages. Our results demonstrate that scutellarin can inhibit the invasive potential of HCC cells and the mechanism for the same involves the suppression of JAK2/STAT3 pathway signalling and also EMT in HCC cells.

The cytoskeleton has been shown to modulate the movement of tumour cells. Yilmaz and Christofori showed that actin cytoskeleton remodelling corresponds to EMT and thereby regulates cell motility.^15^ In line with this observation, we also show by electron and fluorescence microscopy, that in HCC cells, marked cytoskeletal reshaping occurs after scutellarin treatment.

Many studies have shown the antitumour effects of *Scutellaria barbata* extracts before, however, the extract is a heterogeneous mixture, which makes its further application difficult. Our study has a unique advantage, since we studied the antitumour effect of scutellarin, the main ingredient of *S barbata*. Thus, our findings confer reliability to the existing research. Moreover, we explored the cellular mechanics by visualization of the cytoskeleton structure and F‐actin filaments in HepG2 and MHCC97‐H cells upon scutellarin treatment for the first time.

In conclusion, we observed that scutellarin can potentially inhibit the invasive potential of HepG2 and MHCC97‐H cells in vitro, by possibly remodelling the cytoskeleton. The molecular mechanism behind it might be an inhibition of EMT, which could be attributed to the down‐regulation of JAK2/STAT3 pathway signalling. These findings may provide new ideas for the treatment of liver cancer in clinical settings.

## CONFLICT OF INTEREST

The authors declare no conflict of interest.
